# The Functional Network of the Visual Cortex Is Altered in Migraine

**DOI:** 10.3390/vision5040057

**Published:** 2021-11-18

**Authors:** Jie Huang, Arnold Wilkins

**Affiliations:** 1 Department of Radiology, Michigan State University, East Lansing, MI 48824, USA; 2 Department of Psychology, University of Essex, Colchester CO4 3SQ, UK; arnold@essex.ac.uk

**Keywords:** migraine, migraine aura, fMRI, FAUPA, visual cortical functional network

## Abstract

Migraine is a common neurological disorder characterized by recurrent episodes of headache, frequently accompanied by various reversible neurological disturbances. Some migraine patients experience visually triggered migraine headache, and most attacks of migraine with aura are associated with the disturbance of vision and photophobia, suggesting an abnormal neural activity in the visual cortex. Numerous studies have shown a large cortical hemodynamic response to visual stimulation and an altered intrinsic visual functional connectivity network in patients with migraine. In this interictal study, we applied a novel data-driven method with fMRI to identify the functional network in the visual cortex evoked by visual stimulation and investigated the effect of migraine on this network. We found that the distribution of the functional network along both the ventral and dorsal visual pathways differed between migraine patients and non-headache healthy control participants, providing evidence that the functional network was altered in migraine between headaches. The functional network was bilateral in the control participants but substantially lateralized in the migraine patients. The results also indicated different effects of colored lenses on the functional network for both participant groups.

## 1. Introduction

Migraine is a common neurological disorder characterized by recurrent episodes of headache, frequently accompanied by various reversible neurological disturbances. Disturbance of vision and photophobia are associated with most attacks of migraine with aura (MwA), and the aura is most likely to be visual [1]. Between migraine attacks, perceptual illusions and visual discomfort in response to stressful patterns are reported by most migraine patients and those with frequent headache [2,3,4]. Bright light, flickering light, and certain visual patterns can trigger a migraine attack [5,6]. These effects indicate an altered visual cortical function in migraine. Blood oxygenation level dependent (BOLD) functional magnetic resonance imaging (fMRI) provides a non-invasive neuroimaging tool to study the cortical activation in the human brain evoked by visual stimulation [7,8,9]. Several fMRI studies have shown a dysfunction of the visual network in both MwA and migraine without aura (MwoA) [10]. A large BOLD response in the visual cortex was found in response to visual stimulation in MwA [11,12,13,14]. An increased BOLD response to visual stimulation in the downstream visual network areas and motion perception areas was also found in MwA [15,16]. Altered BOLD responses in both the occipital cortex and brainstem structures during stimulation-triggered visual symptoms and/or migraine attacks have been reported [17,18]. The retinotopic mapping technique has enabled the identification of area V3A as the initiation site of an exercise-induced migraine aura and its subsequent spreading through the cortical visual areas [19]. Heterogenous migraine aura symptoms (e.g., visual scotoma and flickering, black and white spots, white spots and flickering lines, etc.) were found to be associated with visual cortical BOLD responses from area V1 to V4 [20]. The abnormal BOLD responses of MwoA patients to visual stimulation also suggested a dysfunction of the visual network in this subgroup of migraine [21].

The intrinsic activity of the human brain maintains its general operation at rest [22]. Measured with the resting-state fMRI (rs-fMRI), this on-going activity exhibits a large spontaneous temporal fluctuation with a high degree of spatial organization across the entire brain [23,24]. Functional connectivity (FC) analyses of rs-fMRI demonstrate the existence of large-scale brain FC networks, including a visual FC network [25]. Altered FC networks during the resting-state have been observed during and between migraine attacks in both MwA and MwoA [26,27,28,29,30,31,32,33,34,35,36,37,38]. In comparison to the non-headache control group, the FC between the anterior insula and occipital areas, including area V3A, was reduced, but the FC within the visual network was increased in interictal MwA but not in MwoA, respectively [30,32]. During migraine attacks, for MwA patients, the FC between area V5 and the lower middle frontal gyrus in the symptomatic hemisphere was increased [34].

The intrinsic visual FC network is different from the visual stimulation-evoked FC network, although these two networks are related to each other. The latter reflects the information-processing network specific to the stimuli [39]. We recently reported the discovery of functional areas of unitary pooled activity (FAUPAs) with fMRI that provided a novel method to identify task-evoked FC networks [40]. A FAUPA is defined as an area in which the temporal variation of the activity is the same across the entire area, i.e., the pooled activity is a unitary dynamic activity. New techniques were developed to identify FAUPAs involving an iterative aggregation of voxels dependent upon the inter-correlation of their signal time courses [41]. The determination of a FAUPA is objective and automatic with no requirement of a priori knowledge of the BOLD response induced by the activity. This novel data-driven method enables us to identify task-associated FAUPAs and to determine task-evoked functional networks across the whole brain [40,42]. We recently applied this novel FAUPA method to identify both the face-evoked visual-processing network and intrinsic visual FC network, and to investigate the effect of progressive Alzheimer’s disease on these networks [43,44]. Here, we used the method to investigate the functional network in migraine evoked by visual stimulation. To do so, we first identified a stimulation-associated FAUPA in the primary visual area V1, and then used this FAUPA’s signal time course as the reference function to determine the visual-processing network. We were then able to compare the visual functional network between migraine patients and non-headache control participants, revealing substantial differences. 

## 2. Materials and Methods

This study is a report of a re-analysis of the fMRI images from a previous study [14] using the novel FAUPA technique. The earlier study identified the stimulus-evoked BOLD activation in visual areas of V1, V2, V3, V3A, and V4. The activation when precision ophthalmic tinted lenses (POTs) were worn in spectacles was compared with that when gray and control color lenses were worn. The present study applied the FAUPA method to identify and compare the stimulation-evoked visual cortical functional network in migraine patients with that in non-headache control participants.

Participants: eleven migraine patients (7 MwA and 4 MwoA, 9 female, aged 29–48 years, mean 40.3, SD 6.3) satisfied the inclusion and exclusion criteria for the fMRI study and completed the study. The diagnosis of migraine was based on the criteria of the International Headache Society [45]. The inclusion and exclusion criteria for the migraine patients as well as their demographics are presented in our previous study [14]. Eleven sex- and age-matched non-headache healthy control participants (9 female, aged 30–49 years, mean 39.3, SD 5.9) also participated in the study. The University Institutional Review Board at Michigan State University approved the study (IRB# 07-098, 13 February 2007), and written informed consent forms were obtained from all participants prior to the study. All methods were performed in accordance with the Institution’s relevant guidelines and regulations. 

**Figure 1:**
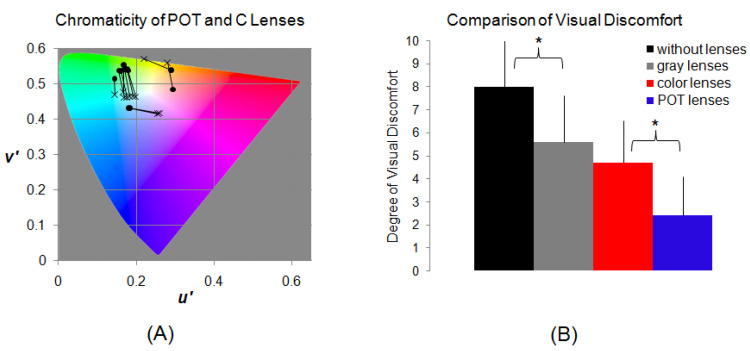
(**A**): CIE 1976 UCS diagram showing the chromaticities of the POTs and the control C lenses used by the eleven migraine patients and their control participants in the fMRI study. The chromaticity of each POT is marked by a solid circular point, and a line connects the point to the chromaticity of its paired control C lens (cross). (**B**): The effects of the POT, the control C, and gray (G) lenses in reducing visual discomfort in the migraine patients relative to those without lenses when viewing the stressful pattern out of doors in direct sunlight. The degree of visual discomfort was self-scored using a 0 to 10 scale, with 0 representing no visual discomfort and 10 representing severe visual discomfort. Reproduced with permission from [14]. (For further details, refer to [14]). The reduction with the POT lenses was significantly larger than that with the coloured lenses (*t*-test, *p* = 0.005), marked with *.

Precision ophthalmic tint and control lenses: prior to the fMRI study, each migraine patient was assessed using the Intuitive Colorimeter (Cerium Visual Technologies, Kent, UK) to obtain an optimal hue and saturation (chromaticity) of light that maximized visual comfort and reduced any perceptual distortion of text [46,47]. This optimal setting was used to prescribe a POT for the patient that would match the patient-selected shade of color (chromaticity) under white lighting. Two additional lenses were chosen as control lenses: one was gray (G) and the other was a control color (C). All three lenses had similar photopic transmission. The saturation of both colored lenses (POT and C) was similar. The chromaticity of the C lens differed from that of the POT by an average of 0.07 in the 1976 CIE UCS diagram [48]. A difference of 0.07 is more than twice the standard deviation when colorimeter assessments are repeatedly undertaken [49], and it is sufficient to remove the effects on symptoms that have been reported [50,51] and also to eliminate the increase in reading speed that occurs with some groups when wearing lenses with their POT [52]. All lenses were made of CR39 resin, which does not affect MRI signals. Figure 1 illustrates the distribution of the chromaticity of these POT and C lenses in the CIE UCS diagram and the effects of these lenses in reducing visual discomfort in the migraine patients. (For further details, refer to [14]). 

**Figure 2:**
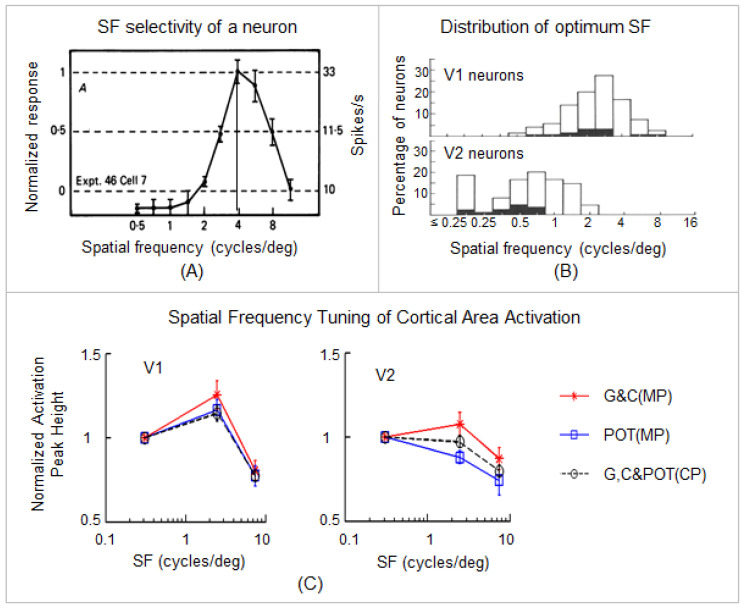
The effects of the colored lenses on the SF-tuned BOLD response in area V1 and V2. (**A**): A representative SF tuning curve shows a band-pass response with an optimum SF (the vertical line). The majority of curves for macaque neurons in V1 (91%) and V2 (100%) are similar to this curve. (**B**): The distribution of optimum SF for V1 (top) and V2 (bottom) neurons. The difference between the means of the distributions (V1 = 2.2 cycles/deg; V2 = 0.65 cycles/deg) is statistically significant (*p* < 0.001). The overlap between the two distributions is principally in the 0.7–2 cycles/deg range. (**A**,**B**) are reproduced with permission from [53]. (**C**): SF-tuned cortical area activation in V1 and V2. The dashed lines represent the mean peak heights of cortical area activation with the three lenses for the control participants (CP). The red solid lines represent the mean peak heights of cortical area activation with the G and C lenses for the migraine patients (MP). The blue solid lines represent the peak heights of cortical area activation with the POT lenses for the MP. Error bars indicate the standard errors of the means. (**C**) is reproduced with permission from [14]. (For further details, refer to [14]).

Image acquisition: functional brain images covering the whole occipital cortex were acquired on a GE 3T Signa^®^ HDx MR scanner (GE Healthcare, Waukesha, WI, USA) with an 8-channel head coil using a gradient echo-planar-imaging pulse sequence (TE/TR = 45.3/2000 ms, flip angle 80°, FOV 22 cm, matrix 96 × 96, slice thickness 3.0 mm, number of slices 20, and first 5 time points discarded). The visual stimuli consisted of three vertical black and white striped patterns (square-wave luminance profile) with three spatial frequencies (SFs) of 0.31 cycles per degree (cpd) (non-stressful), 2.5 cpd (stressful), and 7.9 cpd (less stressful), respectively. The high SF 7.9 cpd enabled us to examine the SF-tuning of the BOLD response across the visual areas [14] (see Figure 2). The stimuli were 10° high and 13° wide in visual angle and were presented via a 32-inch LCD monitor (Salvagione Design, Sausalito, CA, USA) placed at the back of the scanner. The stimulation paradigm consisted of 12 10 s long stimulation blocks interleaved with 12 24 s fixation blocks (rest periods); each SF was presented four times in a pseudo-random order, resulting in a total of 204 volume images per anatomic location for each scan. Each participant had three visual activation scans; the participant had G lenses for the first scan and then had either POT or C lenses for the next two scans (counter-balanced across the participants). Each control participant (matched in age and sex to a migraine patient) was tested with the same three lenses as the migraine patient in the same order. During the scan, the subject’s attention was controlled by randomly changing the fixation mark at the center of the visual field from square to cross or vice versa, and the subject responded by pressing a button on an MRI-compatible keypad when a change occurred. (For further details, refer to [14]).

Image pre-processing: a standard image pre-processing procedure was performed using the AFNI (analysis of functional neuro images) software [40,54]. It included removing spikes, slice-timing correction, motion correction, spatial filtering with a Gaussian kernel with a full-width-half-maximum of 3.0 mm, computing the mean volume image, band-passing the signal intensity time course to the range of 0.009 Hz–0.08 Hz, and computing the relative signal change ΔS (%) of the band-passed signal intensity time course for each of the three scans of each participant [40]. ΔS = [S(t) − S_0_] × 100/S_0_ (%), where S(t) is the signal time course and S_0_ is the mean of S(t). Further image analysis was carried out using Matlab-based software algorithms developed in-house.

Identification of stimulation-associated FAUPAs in area V1: a statistical model and algorithms were developed and implemented in Matlab to identify FAUPA [40]. The determination of a FAUPA is based on the assumption that the signal time course is the same across the entire area within a FAUPA, and its determination involved an iterative aggregation of voxels dependent upon the inter-correlation of their signal time courses. The determination consisted of two major procedures: (a) the algorithm, with an initial statistical criterion, identified a stable region-of-interest (ROI) in which the signal time courses of all voxels showed a similar temporal behavior; and (b) using a second statistical criterion, the algorithm then compared the temporal behavior of the signal time course of the voxels within the ROI with those bordering it to determine whether this stable ROI satisfied the condition of being a FAUPA. (For further details, refer to [40]). FAUPAs were first identified for each lens type and each participant. The signal time course of a stimulation-associated FAUPA in area V1 should resemble the time-locked visual stimulation-induced BOLD signal changes that followed the stimulation paradigm. For each participant, for the three lens types, we identified three stimulation-associated FAUPAs in area V1 that had an overlapped common area (voxels) shared by all three FAUPAs. Because the signal time course is the same across the entire area within each FAUPA, the overlapped common area served as an unbiased seed for all three lens types in conducting a seed-based FC analysis [55]. 

The stimulation-evoked visual cortical functional network: with the selected stimulation-associated FAUPA in area V1 for each lens type and each participant, its signal time course, averaged over all voxels within the FAUPA, was used as the reference function to compute its correlation coefficient (R) with the signal time courses of all voxels so as to yield an R map in the 3-dimentional coordinate system of the MRI scanner (the so-called original space). This gave a separate R map for each lens type and each individual, 66 R maps in all. Each R map was an independent measure; it measured the correlation of the cortical activity between each area within the brain and the stimulation-associated FAUPA in area V1, i.e., the FC that reflects the strength of the co-activity across the brain with the FAUPA’s activity for that lens type and that individual. Then, using AFNI, we converted all R maps to a standard template space (icbm452) for group analysis. In this standard template space, for each participant group and each lens type, the group-mean R map was first computed, averaged over all participants within that group. This resulted in 6 independent group-mean R maps in all (i.e., three lens types × two participant groups). For each group and each lens type, a threshold was applied at R > 0.5 (N = 204, *p* = 2.6 × 10^−14^) to yield a stimulation-evoked visual cortical functional network for that group and that lens type, 6 independent visual functional networks in all. To analyze these networks, the grand-common network (GCN) shared by all three lens types and two participant groups was identified from the voxels shared by all six networks. This GCN was an essential part of the visual functional network that was independent of the lenses and migraine. To examine the effect of migraine on the visual functional network, separately for each participant group, the lens-common network (LCN) shared by all three lens types was determined from the voxels shared by the three independent networks for that group (i.e., separately for migraine and control groups). This gave two LCNs, one for the patient group and the other for the control group. Each LCN was used to determine the part that was distinct from the GCN, i.e., the voxels within the LCN that were unique for that participant group. These two distinct parts for the two participant groups were then used as masks to analyze whether these two LCNs were different from each other with respect to both the spatial extent and FC. Finally, for each participant group, the part of each of the three networks that was distinct from that group’s LCN was identified, separately for each lens type, and used as a mask (voxels of interest) to examine the effects of the three lenses on the visual functional network for that participant group. As the range of R is from −1 to 1, for group statistical tests of these R values, R was converted to Z through Fisher’s Z-transformation, i.e., Z = ln[(1 + R)/(1 − R)]/2 with the range [−∞, ∞], resulting in an improved normality. All statistical tests were based on their corresponding Z-scores. The normality of each measured sample was tested with the Kolmogorov–Smirnov (KS) test and reported if the sample’s distribution was significantly different from the normal distribution.

## 3. Results

### 3.1. Identification of Stimulation-Associated FAUPAs

**Figure 3:**
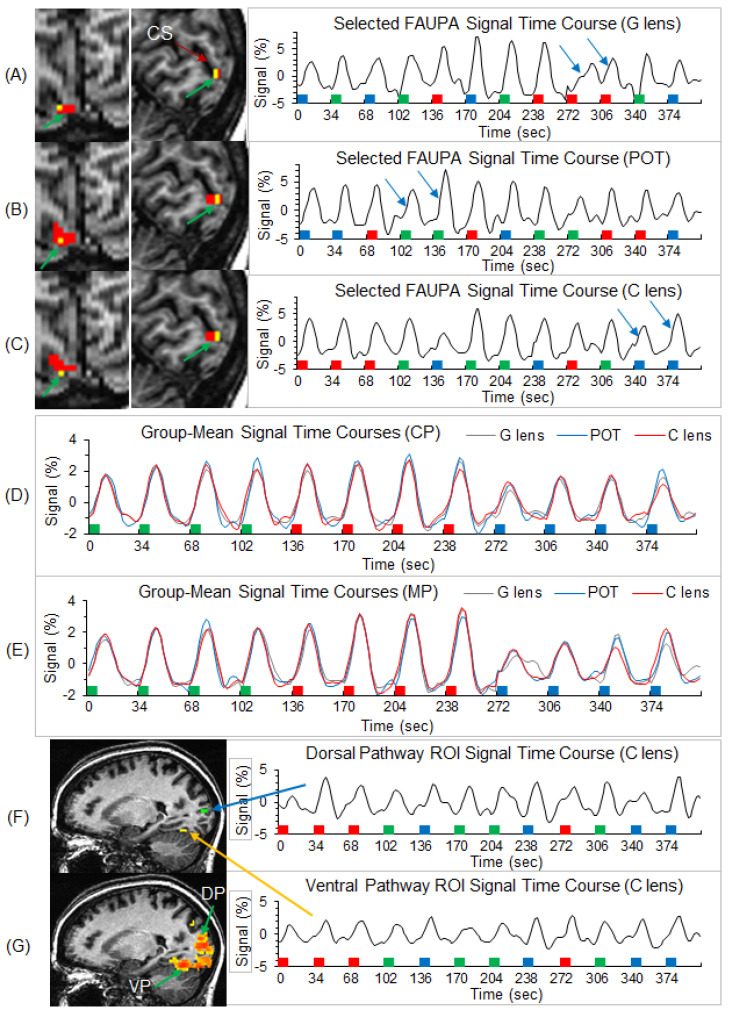
Illustration of the three selected stimulation-associated FAUPAs in area V1 in the original space and their corresponding signal time courses for a representative participant, and the group-mean signal time courses of the selected FAUPAs for the three lens types and two participant groups. Top three panels (**A**–**C**): the green arrow points to small red clusters in the left images, which represent the selected stimulation-associated FAUPAs in area V1 for the three lens types, respectively. The yellow voxels represent the overlapped area across these three FAUPAs. For each FAUPA, the right plot shows its signal time course averaged over all voxels within that FAUPA. For each time course, the magnitude represents the percentage signal change relative to the mean signal of the time course. In each plot, the two blue arrows indicate a noticeable variation in the BOLD response for the same stimulus. The green, red, and blue bars represent the onset and duration of the three striped patterns with SF of 0.31, 2.5, and 7.9 cpd, respectively. CS: calcarine sulcus; (**G**): gray; POT: precision ophthalmic tint; (C): color. Middle two panels (**D**,**E**): for each of the two participant groups, the plot shows the group-mean signal time courses of the selected FAUPAs for the three lens types, respectively. Note that, as the stimuli were presented in a pseudo-random order in each scan, for computing the group-mean signal time course for each lens type and each group, the signal time course of each selected FAUPA was first sorted according to the SFs and then averaged over all participants within the group. CP: control participants; MP: migraine patients. Bottom two panels (**F**,**G**): illustration of the visual stimulus-processing network along both the dorsal and ventral pathways for the representative participant. One green region of interest (ROI) with 4 voxels was placed on the dorsal pathway (DP), and one yellow ROI was placed on the ventral pathway (VP) (top left image). The right two plots show the ROI-mean signal time course for these two ROIs indicated by the blue and yellow arrows. The colored clusters in the bottom left image show the R map with threshold R > 0.5, i.e., the visual stimulus-processing network.

Individual-level stimulation-associated FAUPAs were identified in area V1 (Figure 3). For each participant, the three selected FAUPAs (one for each of the three lens types) had an overlapped area as illustrated in the top three panels in Figure 3A–C. (For consistency, all stimulation-associated FAUPAs were located in area V1 of the right cerebral hemisphere of all the participants.) The stimulation-evoked signal changes were conspicuous for each of the three lens types, with variation from trial to trial, from lens type to lens type, and from participant to participant (the right three plots in Figure 3A–C). The group-mean signal time course reflected the stimulation paradigm and showed similar behavior for the three lens types and two participant groups (Figure 3D,E). In area V1, the stressful striped pattern with SF 2.5 cpd evoked the signal changes with the largest peak (the middle four signal changes in Figure 3D,E), followed by the non-stressful pattern with SF 0.31 cps (the first four signal changes), and then the less stressful high SF 7.9 cpd (the last four signal changes), showing the expected SF-tuning of the BOLD response in area V1 [14].

### 3.2. Stimulation-Evoked Visual Cortical Functional Network

**Figure 4:**
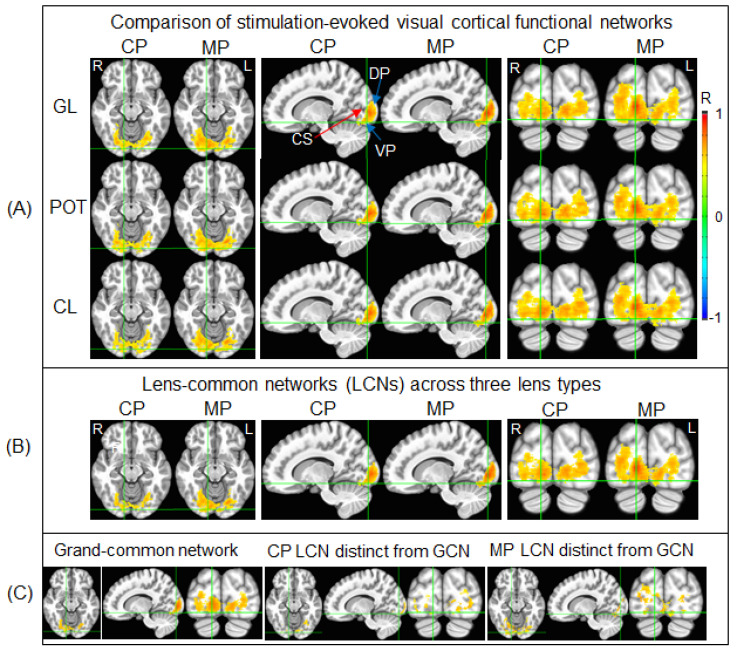
Illustration of the stimulation-evoked visual cortical functional networks. (**A**): For each participant and each lens type, a Pearson correlation coefficient (R) map was first computed using the signal time course of the selected stimulation-associated FAUPA in area V1 (Figure 3A) as the reference function; then, for each group, in the standard space, the group-mean R map was computed with threshold R > 0.5 to yield the stimulation-evoked visual cortical functional network for that group. The stimulation evoked the cortical representation of the foveal vision in V1 near the occipital pole with a narrowed width of the activated area along the calcarine sulcus (CS) that represents the eccentricity axis of the visual field from the center to periphery, consistent with the small visual field of the stimuli (10° high and 13° wide). As expected, the activation propagated along both the dorsal pathway (DP) and ventral pathway (VP). CP: control participants; MP: migraine patients; GL: gray lens; POT: precision ophthalmic tint; CL: color lens; R: right; L: left. (**B**): For each group, the lens-common network (LCN) shared by all three functional networks of the three lens types. (**C**): The grand-common network (GCN) shared by the two LCNs (left), the CP’s LCN distinct from the GCN (middle), and the MP’s LCN distinct from the GCN (right), respectively.

For each lens type, Figure 4A illustrates the stimulation-evoked functional networks identified for the control participants and migraine patients, respectively. As can be seen, V1 was activated and the activation propagated along both dorsal and ventral pathways, showing that the visual information was processed along both these pathways. Within each participant group, the stimulation-evoked functional network was similar across the three lens types, reflected in its corresponding LCN shown in Figure 4B. This LCN comprised the GCN and an additional distinct network, different for the control participants and migraine patients. The distinct components differed substantially between the control participants and migraine patients. These differences are evident when comparing the GCN and the two distinct functional networks shown in Figure 4C.

### 3.3. Altered Visual Functional Network in Migraine

**Figure 5:**
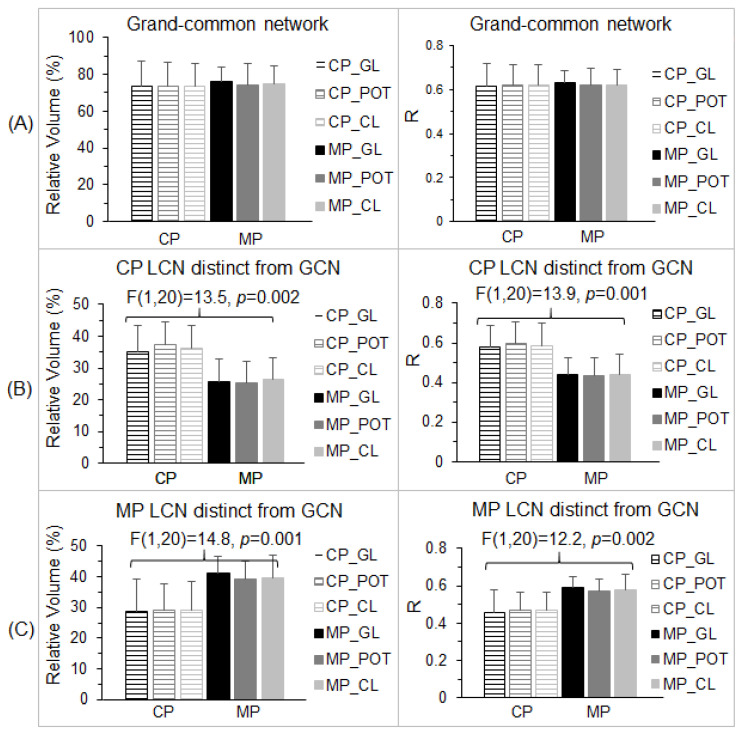
Group comparisons of the two visual cortical functional networks for the gray lens (GL), precision ophthalmic tint (POT), and color lens (CL). (**A**): For the grand-common network (GCN) (Figure 4C, left), the group-mean relative volume (left) and R values (right) were at the same level for all three lens types and two groups of control participants (CP) and migraine patients (MP). (**B**): For the part of the lens-common network (LCN) of the CP distinct from the GCN (Figure 4C, middle), the group-mean relative volume (left) and R values (right) of the CP were at the same level for all three lens types. Although the group-mean relative volume and R values of the MP were at the same level for all three lens types, they were significantly smaller than that of the CP. (**C**): For the part of the LCN of the MP distinct from the GCN (Figure 4C, right), the group-mean relative volume and R values of the MP were at the same level for all three lens types. The group-mean relative volume and R values of the CP were at the same level for all three lens types, but significantly smaller than those of the MP. The error bars indicate the standard deviations.

The GCN (Figure 4C, left) characterized the essential part of the stimulation-evoked visual functional network that was independent of the lenses and migraine. Consequently, we should expect no difference in both the total number of activated voxels and the strength of the FC within this GCN between the control participants and migraine patients for all three lens types. We undertook two separate analyses to verify this expectation. First, for each lens type and each participant group, within the GCN, we computed the total number of voxels with R > 0.5 (i.e., activated voxels) and its ratio to the GCN’s total number of voxels for each lens type and each participant. (The GCN’s total number of voxels was 15,507 and its corresponding total volume was 15.507 cm^3^.) For each participant group, the group-mean relative volume was computed for each lens type, and this relative volume was similar for all three lens types and two participant groups (Figure 5A, left). A two-way repeated measures ANOVA with the participant group as the between-participant factor and the three lens types as the within-participant factor showed no main effect of the group, no effect of the lens type, and no interaction (*p* > 0.74 in all cases). Second, for each lens type and each participant, using that individual’s R map for that lens type, we computed a GCN-mean R (i.e., the strength of FC) averaged over all the voxels within the GCN for that participant and that lens type. For each participant group, the group-mean R was computed for each lens type. This group-mean R was similar for all three lens types and two participant groups (Figure 5A, right). The two-way repeated measures ANOVA also showed no main effect of the group, no effect of the lens type, and no interaction (*p* > 0.91 in all cases). These results demonstrated that, within the GCN, there was no difference in both the spatial extent of the activated voxels and the strength of the FC between the control participants and migraine patients for all three lens types.

**Figure 6:**
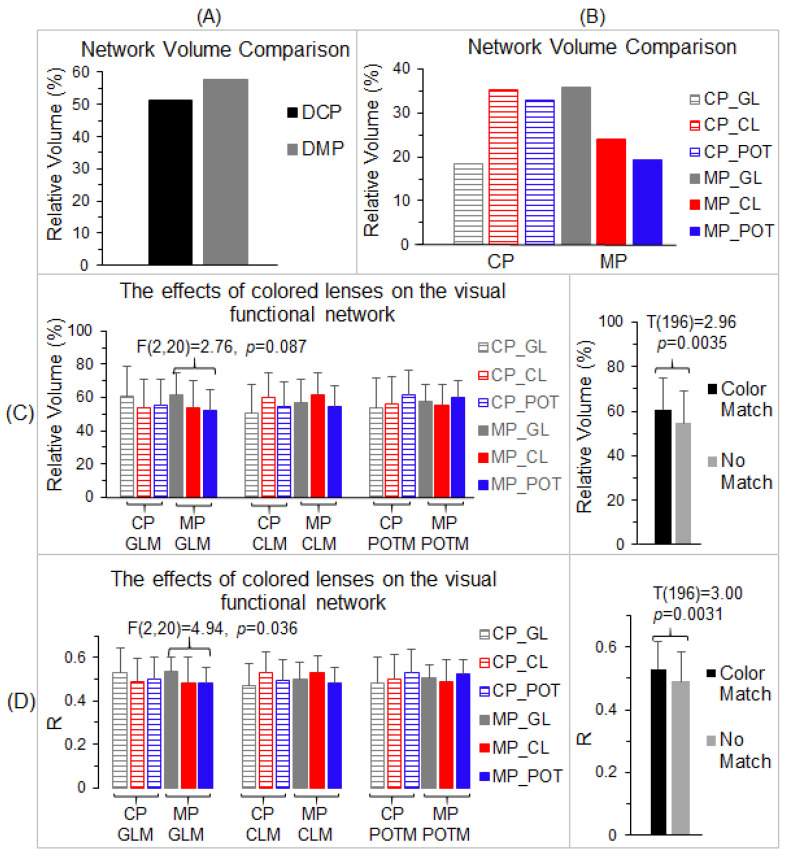
Comparison of the relative network volume and the effects of color on the visual functional network. (**A**): The relative volume of the control participants’ (DCP) lens-common network (LCN) distinct from the grand-common network (GCN) and of the migraine patients’ (DMP) LCN distinct from the GCN, respectively. (**B**): For each participant group, the relative volume of the visual functional network of each lens type distinct from the LCN of that group. (**C**): Comparison of the relative volume of voxels with R > 0.5 within each mask for the three lens types using each of the six distinct visual functional networks in B as a mask (left). Right: group-mean of relative volume of activated voxels for two groups; one with the color-matched (Color Match) between the lens color and the color of mask, and the other group in which the lens color does not match the color of mask (No Match). (**D**): Comparison of the mask-mean R values for the three lens types using each of the six distinct visual functional networks in B as a mask (left). Right: group-mean R for the two Color Match and No Match groups. CP: control participants; MP: migraine patients; GL: gray lens; CL: color lens; POT: precision ophthalmic tint; and M: mask. The error bars indicate the standard deviations.

To compare the visual cortical functional network between the control participants and migraine patients, the total number of voxels within the LCN was compared with the number in the GCN for each participant group. Using the volume of the GCN as the reference, the relative volume of the part of the LCN distinct from the GCN (Figure 4B) was found to be 51.4% for the control participants and 57.6% for the migraine patients, respectively, roughly half the size of the GCN (Figure 6A). Although the size of the LCN was similar for both the control group and migraine group, *less than two-thirds of the LCN was shared by them*, showing an extensively altered visual functional network in migraine. To verify this altered visual functional network, for each part of the LCN distinct from the GCN, we compared the total number of activated voxels and the strength of the FC within that part among the three lens types for each participant group and between the two participant groups.

We first examined the LCN of the control participants. For the part *distinct* from the GCN (Figure 4C, middle), and using this part as a mask, we computed the total number of voxels with R > 0.5 within the mask and its relative volume to the GCN for each participant and each lens type. For each participant group, the group-mean relative volume was computed for each lens type. This relative volume was similar for all three lens types within each participant group but substantially larger for the control participants in comparison to the migraine patients (Figure 5B, left). The two-way repeated measures ANOVA showed a significant main effect of the participant group [F(1,20) = 13.5, *p* = 0.002] but no effect of the lens type and no interaction (*p* > 0.74 in all cases). The significant reduction in the relative volume for the migraine patients showed that the visual stimulation activated significantly fewer voxels in this distinct part in comparison to that for the control participants. To examine the strength of the FC within this mask, we computed the mask-mean R averaged over all the voxels within the mask for each participant and each lens type, and then the group-mean R for each lens type. The group-mean R value of the control participants was at the same level for all three lens types (Figure 5B, right), and this R value was also at the same level as that for the GCN (Figure 5A, right), consistent with the LCN that was evoked by the visual stimulation for the control participants. For the migraine patients, however, the group-mean R value was substantially smaller in comparison to that of the control participants independent of the lens types (Figure 5B, right). The two-way repeated measures ANOVA showed a significant main effect of the participant group [F(1,20) = 13.9, *p* = 0.001] but no effect of the lens type and no interaction (*p* > 0.8 in all cases). [One sample’s distribution was significantly different from the normal distribution (the KS test, *p* = 0.048)]. This significantly reduced FC showed that the distinct part was not evoked by the visual stimulation for the migraine patients.

In the same way, we examined the LCN of the migraine patients. For its part distinct from the GCN (Figure 4C, right), we computed the total number of activated voxels within this part and its volume relative to the GCN for each participant and each lens type. The group-mean relative volume was similar for all three lens types within each participant group but substantially larger for the migraine patients in comparison to the control participants (Figure 5C, left). The two-way repeated measures ANOVA showed a significant main effect of the participant group [F(1,20) = 14.8, *p* = 0.001] but no effect of the lens type and no interaction (*p* > 0.85 in all cases). The significant reduction in the relative volume for the control participants showed that the visual stimulation activated significantly fewer voxels in this distinct part in comparison to that for the migraine patients. To examine the strength of the FC within this mask, we computed the mask-mean R for each participant and each lens type. The group-mean R value of the migraine patients was at the same level for all three lens types (Figure 5C, right), and this R value was at the same level as that for the GCN (Figure 5A, right), consistent with the LCN that was evoked by the visual stimulation for the migraine patients. For the control participants, however, the group-mean R value was substantially smaller in comparison to that of the migraine patients independent of the lens types (Figure 5C, right). The two-way repeated measures ANOVA also showed a significant main effect of the participant group [F(1,20) = 12.2, *p* = 0.002] but no effect of the lens type and no interaction (*p* > 0.7 in all cases). This significantly reduced FC showed that the distinct part was not evoked by the visual stimulation for the control participants. Overall, the combined results for the two distinct parts of the LCN from the GCN consistently demonstrate a significantly altered visual cortical functional network in the migraine patients.

**Figure 7:**
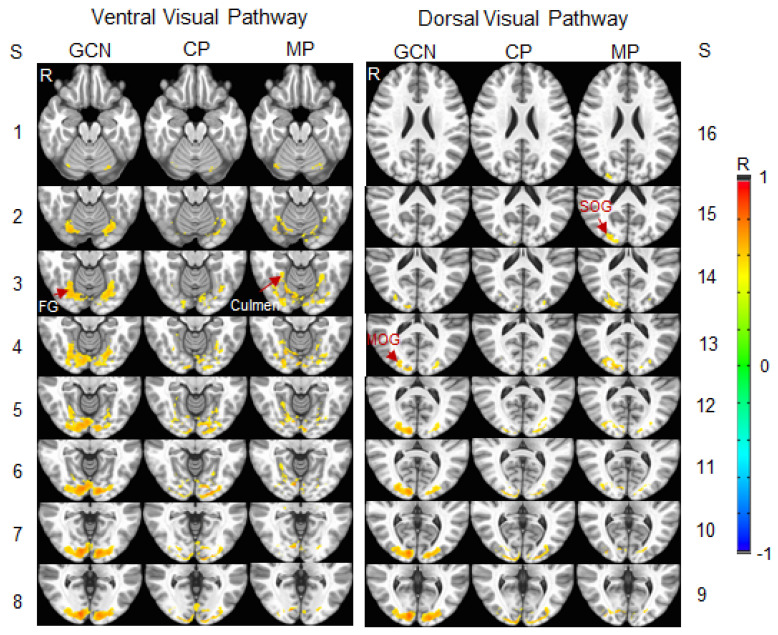
Illustration of the stimulation-evoked visual cortical functional network for the migraine patients (MP) versus the control participants (CP). 1st & 4th columns: the grand-common network (GCN) shared by both networks of the two participant groups in ventral and dorsal visual pathways; 2nd & 5th: CP network distinct from the GCN; and 3rd & 6th: MP network distinct from the GCN. Note that, for each of the two distinct networks, the union of that distinct network with the GCN is the visual cortical functional network of that participant group. Slice (S) thickness 1 mm with 2 mm gap between any two adjacent slices. R: right; FG: fusiform gyrus, Brodmann area (BA) 19; Culmen: BA 37; MOG: middle occipital gyrus, BA 18; and SOG: superior occipital gyrus, BA 18.

To compare the visual functional network between the migraine patients and control participants, an R map was obtained by averaging the three lens types for each participant. A group-mean R map was then obtained by averaging all the participants within each participant group. A threshold of R > 0.5 was set on this group-mean R map to yield the visual functional network for each group. To analyze these two networks, three network masks were generated: (1) the GCN shared by both networks; and (2) two networks distinct from the GCN. Figure 7 illustrates these three networks. In the ventral visual pathway, the GCN started in area V1 and reached the fusiform gyrus (BA 19), and the functional network of the migraine group extended to the culmen (BA 37). In the dorsal visual pathway, this GCN reached the middle occipital gyrus (BA 18), and the functional network of the migraine group extended to the superior occipital gyrus (BA 18). For both visual pathways, within the cerebral cortex, the size of the GCN was larger in the right hemisphere in comparison to that in the left hemisphere (Figure 7, first and fourth columns). In contrast, the size of the control group network distinct from this GCN was relatively large in the left hemisphere compared to that in the right hemisphere (Figure 7, second and fifth columns). These results indicated a bilateral visual functional network for the control group because this network is the union of its distinct network with the GCN. The migraine group network distinct from the GCN, however, showed a relatively large size in the right hemisphere compared to that in the left hemisphere (Figure 7, third and sixth columns), demonstrating a lateralized visual functional network in the migraine patients.

### 3.4. The Effects of Color on the Visual Functional Network

For each participant group (control participants and migraine patients), we used the LCN of that group as the reference, and, for each lens type, we computed the relative volume of the part of that lens’ visual functional network distinct from the LCN (Figure 6B). For the control participants, this relative volume was 18.4% for the gray lens, 35.1% for the color lens, and 32.8% for the precision ophthalmic tint, respectively, showing a similar effect of the color lens and precision ophthalmic tint in the control group. (The precision ophthalmic tint was selected by the migraine patients and not by the control participants.) For the migraine patients, the relative volume was 35.9% for the gray lens, 23.9% for the color lens, and 19.2% for the precision ophthalmic tint, respectively, showing a smaller functional network for the precision ophthalmic tint. These results indicate different effects of the colored lenses on the visual functional network of each participant group. Separately, for each lens type and each participant group, the part of its visual functional network distinct from the LCN of that group was identified and used as a mask to examine the effects of the three lenses on that network. This resulted in three masks (i.e., gray lens mask, color lens mask, and precision ophthalmic tint mask) for each participant group, a total of six conditions (three mask types × two participant groups). We undertook two separate analyses to examine these effects. First, for each mask and each participant group, we computed the total number of voxels with R > 0.5 within that mask and its ratio to the total voxel number of the mask for each lens type and each participant, and then a group-mean relative volume for that lens type and that participant group. This resulted in three group-mean relative volumes of activated voxels for each of the six conditions (Figure 6C, left). These relative volume sizes were compared among the three lens types to determine the effect of each lens type on the spatial extent of the visual functional network. The repeated measures ANOVA compared the three lens types for each of the six conditions and showed no significant difference between the lens types in all six analyses [max F(2,20) = 2.76, min *p* = 0.087]. Nevertheless, when the color of the lens type matched the color of the mask type (e.g., the gray lens matched the gray lens mask), the group-mean volume size was the largest one in comparison to the other two lens types (e.g., the color lens and precision ophthalmic tint). This was true for all six conditions. Accordingly, we grouped these volume sizes into two categories: (1) the group with the color of the lens matching the color of the mask and (2) the group in which the color of the lens did not match the color of the mask. The group-mean volume size of the former was significantly larger than that of the latter (two-tailed *t*-test, *p* < 0.004) (Figure 6C, right), showing the dependence of the effect of the lens on the mask.

Second, for each mask and each participant group, we first computed a mean R value averaged over all the voxels within that mask for each lens type and each participant, and then a group-mean R value for that lens type and that participant group. This resulted in three group-mean R values for each of the six conditions (Figure 6D, left). These R values were compared among the three lens types to determine the effect of each lens type on the FC of the visual functional network. The repeated measures ANOVA compared the three lens types for each of the six conditions. There was a significant difference between the lenses in the condition of the gray lens mask for the migraine group [F(2,20) = 4.94, *p* = 0.036] but not for the other five conditions [max F(2,20) = 3.45, min *p* = 0.052]. Again, when the color of the lens type matched the color of the mask type, the group-mean R was the largest compared to the other two lens types, and this was true for all six conditions. These R values were grouped into the same two categories described above, and the group-mean R of the color-matched group was significantly larger than that of the no-match group (two-tailed *t*-test, *p* < 0.004) (Figure 6C, right), showing again the dependence of the effect of the lens on the mask. Overall, these results demonstrate different effects of colored lenses on the visual cortical functional network in the two participant groups.

## 4. Discussion and Conclusions

In this study, the novel FAUPA method described by Huang [40] was used to identify the stimulation-evoked visual functional network and the network was compared between migraine patients and non-headache healthy control participants. The participants viewed black and white striped patterns when wearing lenses of three different colors, and, accordingly, we identified a stimulation-associated FAUPA in area V1 of the right hemisphere for each lens type and each participant. Figure 3A–C demonstrates that, for each lens type, the signal time course of the identified FAUPA followed the visual stimulation paradigm with noticeable variations from trial to trial, characterizing an individual’s response for each stimulus trial. Using the signal time course of a stimulation-associated FAUPA in area V1 as the reference function, a correlation analysis with all the voxels across the brain can yield a visual functional network specific for that stimulation, i.e., a visual stimulus-processing network. Using the signal time course of a stimulation-associated FAUPA in area V1 to identify the visual stimulus-processing network may have advantages over using an expected ideal response in the general linear model [56,57], as demonstrated in our recent study [43]. This is because it takes into account response variations from trial to trial and from participant to participant (Figure 3A–C), potentially yielding an objectively identified visual stimulus-processing network for each individual. It may be particularly important for patients who have difficulty in performing a task, and in patients whose response is atypical.

The visual stimulus-processing network started from area V1 and extended along the dorsal and ventral pathways for all three lens types and two participant groups (Figure 4). The LCN, shared by the three lens types for each participant group, showed a substantial difference between the two participant groups, i.e., less than two-thirds of the LCN were shared by these two groups (Figure 6A). The GCN, shared by all three lens types and two participant groups, played an essential role in this visual stimulus-processing network that was independent of the lens type and participant group, reflected in its similar value for both the relative volume of the activated voxels and the strength of FC for all six conditions (Figure 5A). For each participant group, the part of its LCN distinct from the GCN was evoked by the visual stimulation only for that participant group (Figure 5B,C), and, therefore, that distinct part is specific for that group. For both participant groups, the size of the distinct part of the LCN was more than 50% the size of the GCN (Figure 6A), showing a different visual cortical functional network for the two groups. The network was bilateral in the control participants but substantially lateralized in the migraine patients (Figure 7). How these altered networks relate to migraine pathology remains to be investigated, although visual function is known to be asymmetric in migraine with aura [58]. A recent review of photophobia in migraine has attributed the photophobia to a cortical hyperexcitability [59]. In photosensitive epilepsy, the evident hyperexcitability is often greater in one cerebral hemisphere than in the other [60].

This study showed different effects of colors on the visual cortical functional network for both non-headache control participants and migraine patients. For each participant group, the colored lenses showed no effect on the shared common network across all three lens types, as expected (Figure 5B,C). Each colored lens, however, evoked some additional cortical areas unique for that color in both participant groups (Figure 6B–D). The volume of these color-evoked areas, relative to the volume of the shared common network, ranged from 18.4% to 35.9% for both groups, indicating different effects of the colored lenses on the visual cortical functional network. In addition, in these color-evoked areas, both the relative volume of the activated voxels and the strength of the FC were significantly larger when the color of the lens matched the color of the mask in comparison to when they did not match (Figure 6C,D, right). For each color-evoked area, however, no significant difference in the relative volume of the activated voxels was observed among the three lens types (Figure 6C, left), although a significant difference in the FC was observed in one area (Figure 6D, left). Nevertheless, the functional role of these color-evoked areas in the color-related visual information processing remains to be investigated. As the POT lenses were prescribed for the migraine patients but not for the non-headache control participants, it will be interesting in the future to test the effect of the POT lenses prescribed for non-headache participants on the color-related visual information processing network.

This study also revealed a substantially lateralized visual cortical functional network in migraine patients (Figure 7). The lateralization of the cortical network is quite consistent with the cortical hyper-excitability with which migraine is associated; indeed, in patients with generalized epilepsy, the threshold for a photoparoxysmal response often differs between the two hemispheres [60,61]. The onset of a typical visual aura and its subsequent spreading through the visual field were found to be retinotopically related with the corresponding visual cortical activity changes, and these activity changes occurred across area V3A, V3, V2, and V1 sequentially, showing a network event that was one-to-one related with the visual aura [19]. A recent study showed an increased FC between area V5 and the lower middle frontal gyrus in the symptomatic hemisphere during migraine attacks for MwA patients [34]. The MwA patients experiencing somatosensory, dysphasic, and motor symptoms (the so-called complex auras) showed a significantly higher FC of the left lingual gyrus, within the visual network, and of the right anterior insula, within the sensorimotor network, when compared to both the simple visual MwA and MwoA patients [62]. Whether the lateralized visual functional network is related to unilateral visual auras and/or visually triggered unilateral migraine attacks remains to be investigated.

Our previous study identified visual cortical area activation in response to visual stimulation and examined the effects of the three colored lenses on the activation in those delineated visual areas of the migraine patients [14]. The present study identified the stimulation-evoked visual FC network for each participant group and compared the similarity and difference in these networks between the groups. Neither study, however, investigated the causal relationship of area activation across these visual areas or the dynamically distributed information-processing within the network. The principle of dynamic causal modeling and Granger causality in fMRI timeseries analysis has been reviewed, and they are used to investigate the causal influence (effective connectivity) among area activations within a network or between different networks [63]. Altered Granger causality connection among pain-related intrinsic FC networks in MwoA has been reported [64]. The altered Granger causality of EEG activity was observed between migraine patients and non-headache control participants, and a different pattern of reduced vs. increased Granger causality in basal conditions and during visual stimulation between MwA and MwoA was also reported [65]. It remains to be investigated how to combine the FAUPA method with the dynamic causal modeling or Granger causality analysis to determine the causal relationship of information-processing or connections within and between FAUPA-determined networks.

## Data Availability

Both the original and processed fMRI images plus final research data related to this publication will be available to share upon request with a legitimate reason, such as to validate the reported findings or to conduct a new analysis.
